# High-grade non-intestinal type sinonasal adenocarcinoma with *ETV6::NTRK3* fusion, distinct from secretory carcinoma by immunoprofile and morphology

**DOI:** 10.1007/s00428-023-03587-6

**Published:** 2023-07-06

**Authors:** Natálie Klubíčková, Elaheh Mosaieby, Nikola Ptáková, Aude Trinquet, Marick Laé, Valérie Costes-Martineau, Alena Skálová

**Affiliations:** 1 Department of Pathology, Faculty of Medicine in Pilsen and University Hospital Plzen,, Charles University, Alej Svobody 80, 323 00 Pilsen, Czech Republic; 2 Bioptical Laboratory, Pilsen, Czech Republic; 3 Molecular-Genetic Laboratory, Bioptical Laboratory, Pilsen, Czech Republic; 4 Department of Biology and Medical Genetics, Second Faculty of Medicine, Charles University and Motol University Hospital, Prague, Czech Republic; 5 Department of Pathology, Hopital Gui de Chauliac, Montpellier, France; 6 Department of Pathology, Centre Henri Becquerel, Rouen, France

**Keywords:** Sinonasal, Nasal cavity, non-intestinal-type adenocarcinoma, *ETV6::NTRK3*, Secretory carcinoma, Salivary duct carcinoma

## Abstract

We report 2 cases of high-grade sinonasal adenocarcinoma with a distinct morphological and immunohistochemical phenotype. Albeit histologically different from secretory carcinoma of the salivary glands, both tumors presented here share an *ETV6::NTRK3* fusion. The highly cellular tumors were composed of solid and dense cribriform nests, often with comedo-like necroses in the center, and minor areas with papillary, microcystic, and trabecular formations without secretions, mostly located at the periphery of the lesion. The cells displayed high-grade features, with enlarged, crowded, and often vesicular nuclei with conspicuous nucleoli and brisk mitotic activity. The tumor cells were immunonegative for mammaglobin while showing immunopositivity for p40/p63, S100, SOX10, and GATA3, as well as for cytokeratins 7, 18, and 19. For the first time, we describe 2 cases of primary high-grade non-intestinal type adenocarcinomas of the nasal cavity, distinct from secretory carcinoma by morphology and immunoprofile, harboring the *ETV6::NTRK3* fusion.

## Introduction

Primary sinonasal adenocarcinomas are rare tumors encompassing a wide morphological spectrum, divided into two groups: intestinal-type sinonasal adenocarcinoma (ITAC) and non-intestinal-type sinonasal adenocarcinoma (non-ITAC) subtypes [1]. While ITACs resemble gastrointestinal primary adenocarcinomas, with columnar epithelial structures and interspersed goblet cells, forming papillae, glands, cribriform structures or, in less differentiated cases, solid nests, as well as occasional mucin lakes, non-ITACs show even wider morphological spectrum. Columnar cells forming variable non-gastrointestinal-like glandular structures are seen in low-grade (LG) cases, whereas high-grade (HG) cases usually consist of solid nests with only a few glands and commonly with central comedo-like necrosis, or individual mucin-producing cells infiltrating into the surrounding stroma. Clear-cell change, endowing an appearance of clear-cell renal cell carcinoma, can be observed in some cases of non-ITAC. In addition, SMARCB1-deficient adenocarcinoma was recently recognized and included in the WHO Classification of Tumours [1] as a subtype of “SWI/SNF complex-deficient sinonasal carcinoma.”

With excessive solid areas, the differentiation between HG sinonasal non-ITAC and other poorly differentiated epithelial neoplasms of the salivary gland and non-salivary gland origin might pose a problem. Recent molecular-genetic findings had aided in the subclassification of primary sinonasal carcinomas, prompting the inclusion of a number of new entities in the 5th edition of the WHO Classification of Head and Neck Tumours [1]. The aggressive SWI/SNF complex-deficient sinonasal carcinomas lack the expression of SMARCA4 or SMARCB1 proteins while displaying relatively monomorphic rhabdoid, plasmacytoid, or epithelioid morphology and infiltrative growth into the surrounding tissue [2]. NUT carcinoma is another novel entity with a very poor prognosis, composed of undifferentiated primitive cells with irregular overlapping nuclei and prominent nucleoli, and defined by pathogenic fusions of the *NUTM* gene, most commonly *NUTM::BRD4* [3]. The diagnosis of exclusion of sinonasal undifferentiated carcinoma (SNUC) should be rendered in cases of a high-grade appearing proliferation of relatively monomorphic, sometimes basaloid-looking tumor cells with the evidence of epithelial origin (cytokeratin immunostaining) and absence of any features pointing toward other possible entities, including but not limited to tumors specific for the sinonasal tract as well as neuroendocrine and neuroepithelial neoplasms, melanoma and salivary gland tumors. Up to 80% of SNUC cases were reported to harbor hotspot mutations of the *IDH2* gene [4, 5], while only rare cases displayed *IDH1* gene mutations [5].

Salivary gland tumors might rarely arise from the minor salivary glands located in the sinonasal tract, representing 5–10% of all sinonasal adenocarcinoma cases [6]. Multiple entities in this group are defined by recurrent genetic alterations, such as *MYB::NFIB* and *MYBL1::NFIB* in adenoid cystic carcinoma [7, 8], *MAML2* fusions in mucoepidermoid carcinoma [9], *NR4A3* upregulation by enhancer hijacking in acinic cell carcinoma [10], or *ETV6::NTRK3* in secretory carcinoma [11, 12].

In the context of salivary gland tumors, *ETV6::NTRK3* fusion is specific for secretory carcinoma (SC) [11]. In typical cases, SC is a circumscribed unencapsulated proliferation of lobules separated by fibrous septa, composed of tumor cells with only mild atypia and low-grade nuclear features, growing in solid-microcystic, solid, tubular, follicular, or papillary-cystic patterns, with bluish PASd-positive luminal secretions and low mitotic activity, positive for S100 and mammaglobin while being negative for DOG1 (except for occasional minor staining), p40, and p63 on immunohistochemical examination [11]. High-grade transformation (HGT) of the low-grade SC, albeit rare, is possible, conveying a more aggressive clinical course and poorer outcomes to the patients affected by such tumors [13, 14]. Similar to other salivary gland tumors, SC might rarely arise in the sinonasal location, retaining the typical morphological features described above [12].

In the nasal cavity, three cases of low-grade non-ITAC were reported to harbor the *ETV6::NTRK3* fusion, as well as one case with a less common *ETV6::RET* fusion [15–17]. In addition, one case of tracheal adenocarcinoma with the morphology of sinonasal low-grade non-ITAC and an *ETV6::NTRK3* fusion was reported [18]. Lastly, the fusion has recently been documented in two high-grade salivary gland adenocarcinomas lacking the typical low-grade morphological features of SC [19, 20].

In this report, we present for the first time two cases of primary high-grade sinonasal adenocarcinomas that were initially classified as high-grade sinonasal non-ITAC. Using RNA sequencing, an *ETV6::NTRK3* fusion was detected in both cases. We aim to contribute to unraveling the position of this neoplasm in the classification of head and neck tumors.

## Materials and methods

### Histological and immunohistochemical studies

For conventional microscopy, excised tissues were fixed in formalin, processed routinely, embedded in paraffin (FFPE), cut, and stained with hematoxylin and eosin. Immunohistochemical staining was routinely performed using an automated Ventana BenchMark ULTRA system (Ventana Medical Systems Inc., Tucson, AZ, USA). The primary antibodies used are summarized in Table 1.

**Table 1:** Immunohistochemical examination

Marker	Clone	Company	Dilution	Case 1	Case 2
AR	SP107	Ventana	RTU	−	−
CDX2	DAK-CDX2	Dako	RTU	ND	−
CK14	SP53	Cell Marque	RTU	ND	+
CK18	CD10	Dako	RTU	+	+
CK19	A53-B/A2.26	Ventana	RTU	+	+
CK5/6	D5/16 B4	Dako	1:100	−	−
CK7	OV-TL 12/30	Dako	1:800	+	+
CK8	35βH11	Ventana	RTU	F+	F+
DOG1	SP31	Ventana	RTU	F+	−
GATA3	L50-823	Biocare Medical	1:100	+	+
Her2	HercepTest	Dako	RTU	−	−
Ki-67	MIB-1	Dako	RTU	42%	51%
Mammaglobin	304-1A5	Dako	RTU	−	−
MUC4	1G8	Santa Cruz Biotech	1:100	−	+
NOR1	H-7	Santa Cruz Biotech	1:50	ND	−
p16	R15-A	DB Biotech	1:100	ND	−
p40	DAK-p40	Dako	RTU	F+	+
p63	DAKp63	Dako	RTU	F+	+
PanTrk	EPR17341	Abcam	1:50	+	+
S100	polyclonal	Dako	RTU	+	+
SALL4	6E3	Sigma	1:800	−	ND
SMA	1A4	Dako	RTU	−	−
SMARCA2	polyclonal	Atlas Antibodies	1:100	N	N
SMARCA4	EPNCIR111A	Abcam	1:1000	N	N
SMARCB1	MRQ-27	Ventana	RTU	N	N
SOX10	SP267	Ventana	RTU	+	+
STAT6	YE361	Abcam	1:1000	ND	−
TTF1	SPT24	Biocare Medical	1:50	−	ND

Abbreviations: *F*, focally; *N*, normal expression; *ND*, not done; *RTU*, ready-to-use; +, positive; −, negative

### Next-generation sequencing

For mutation analysis, TruSight Oncology 500 panel, a comprehensive NGS assay on FFPE samples that identifies fusion transcripts, somatic variants, copy number changes, tumor mutational burden, and microsatellite instability was used. NA libraries were created using the TruSight Oncology 500 Kit (Illumina) following the manufacturer’s instructions (KAPA Biosystems, Washington, MA), although we used KAPA FragKit (KAPA Biosystems, Washington, MA) for DNA enzymatic fragmentation. Following the manufacturer’s guidelines, sequencing was conducted using an Illumina NovaSeq6000 sequencer. The OmnomicsNGS analysis program was used for data analysis (DNA variant filtering and annotation) (Euformatics, Finland). Reported variants were filtered retaining variations with coding effects, read depths greater than 50, and variants with allelic frequency >10%, with the removal of benign variants according to the ClinVar database. The remaining collection of variations was visually verified in raw data, and probable artifactual variants were eliminated. The list of genes covered by this panel is available at the product website (https://www.illumina.com/products/by-type/clinical-research-products/trusight-oncology-500.html). In addition, the samples were analyzed using the NGS-based ligation-dependent multiplex RT-PCR assay as described previously [21].

## Case presentation

### Clinical features

**Fig. 1:**
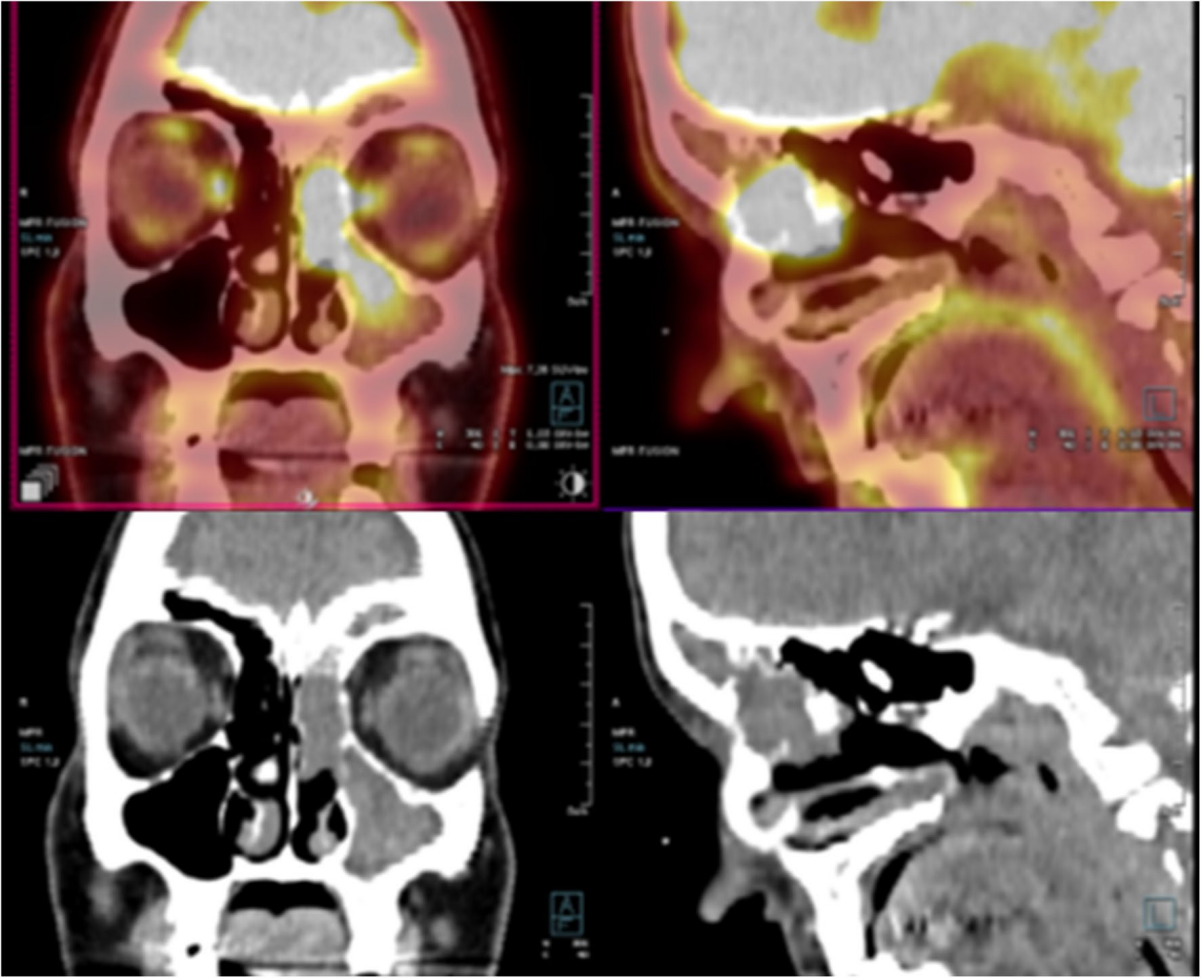
PET/CT imaging study performed in case 1. The tumor filled the left maxillary sinus, extended into the nasal cavity, and infiltrated into the left orbit

In case 1, a 39-year-old female patient presented with nasal obstruction. The tumor filled the left maxillary sinus, extended into the nasal cavity, and infiltrated into the left orbit (Fig. 1). The lesion was staged clinically as cT4a and was inoperable without orbital exenteration. After a diagnostic biopsy, the patient received three cycles of neoadjuvant chemotherapy with docetaxel, carboplatin, and fluorouracil, achieving significant regression of the tumor. Four months after the first diagnosis, the patient underwent a conservative surgical removal of the residual lesion, with a positive posterior surgical margin, prompting the administration of adjuvant proton therapy and chemotherapy with cisplatin. After finishing the treatment, the patient had no signs of disease on clinical examination; however, a CT scan was not yet performed because the case is recent.

A 39-year-old male presented with epistaxis in case 2, caused by a mass in the left nasal cavity, measuring 18 mm in the greatest dimension. After a preliminary biopsy was taken and diagnosed, complementary ethmoidectomy was performed with clear surgical margins. Consequently, the patient received radiotherapy. The patient was alive with no evidence of disease 17 months after the first diagnosis.

### Histopathological features

**Fig. 2:**
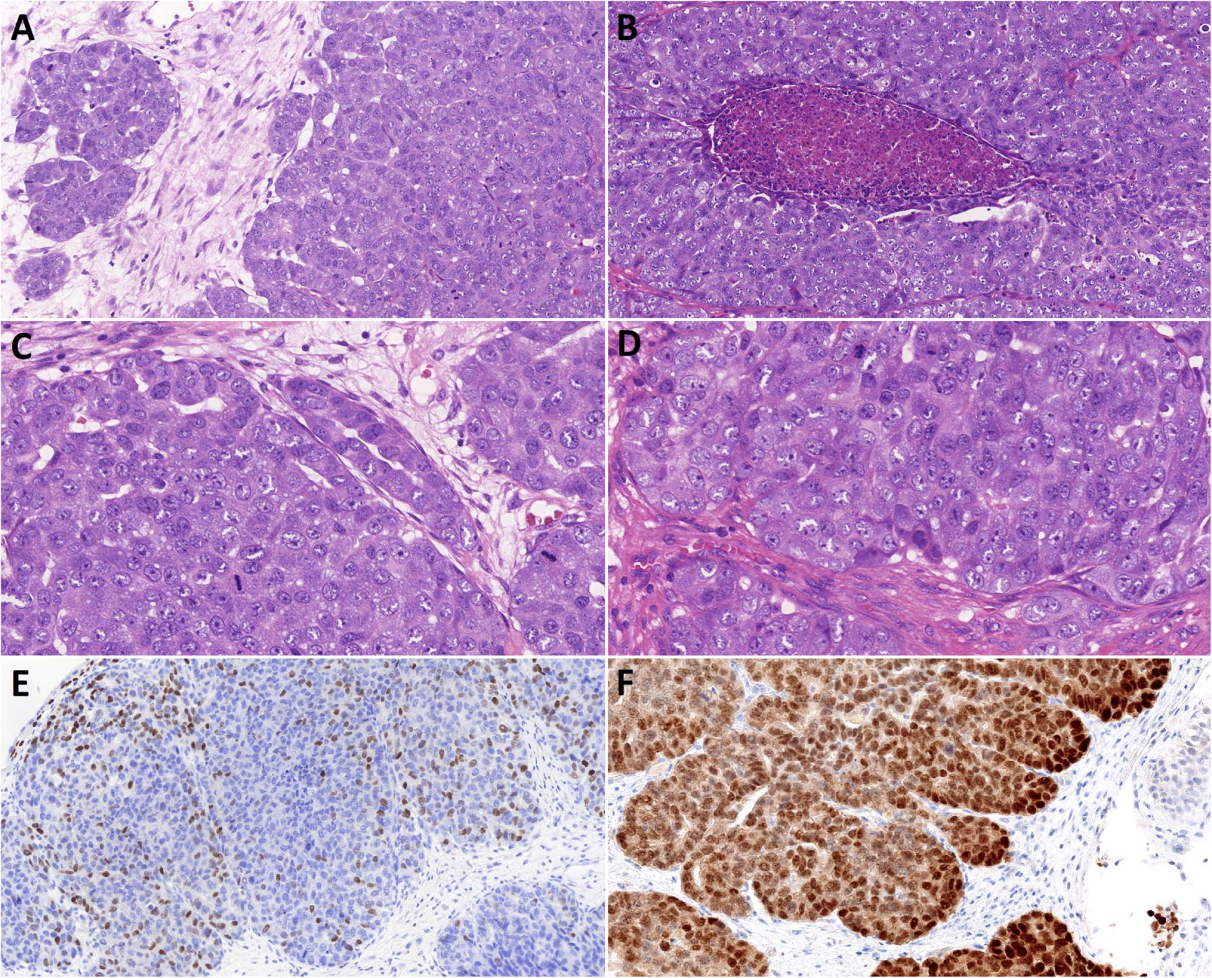
Histopathological features of case 1. **A-B** The tumor grew mostly in solid or dense cribriform nests with central necrosis, displaying high-grade nuclear features and brisk mitotic activity. Focally, especially at the periphery of the tumor, tubular and cribriform formations were observed. **C–D** High-grade cytologic atypia was observed in the tumor cells. **E** Scattered nuclear p63 immunopositivity. **F** Diffuse nuclear PanTrk immunostaining

**Fig. 3:**
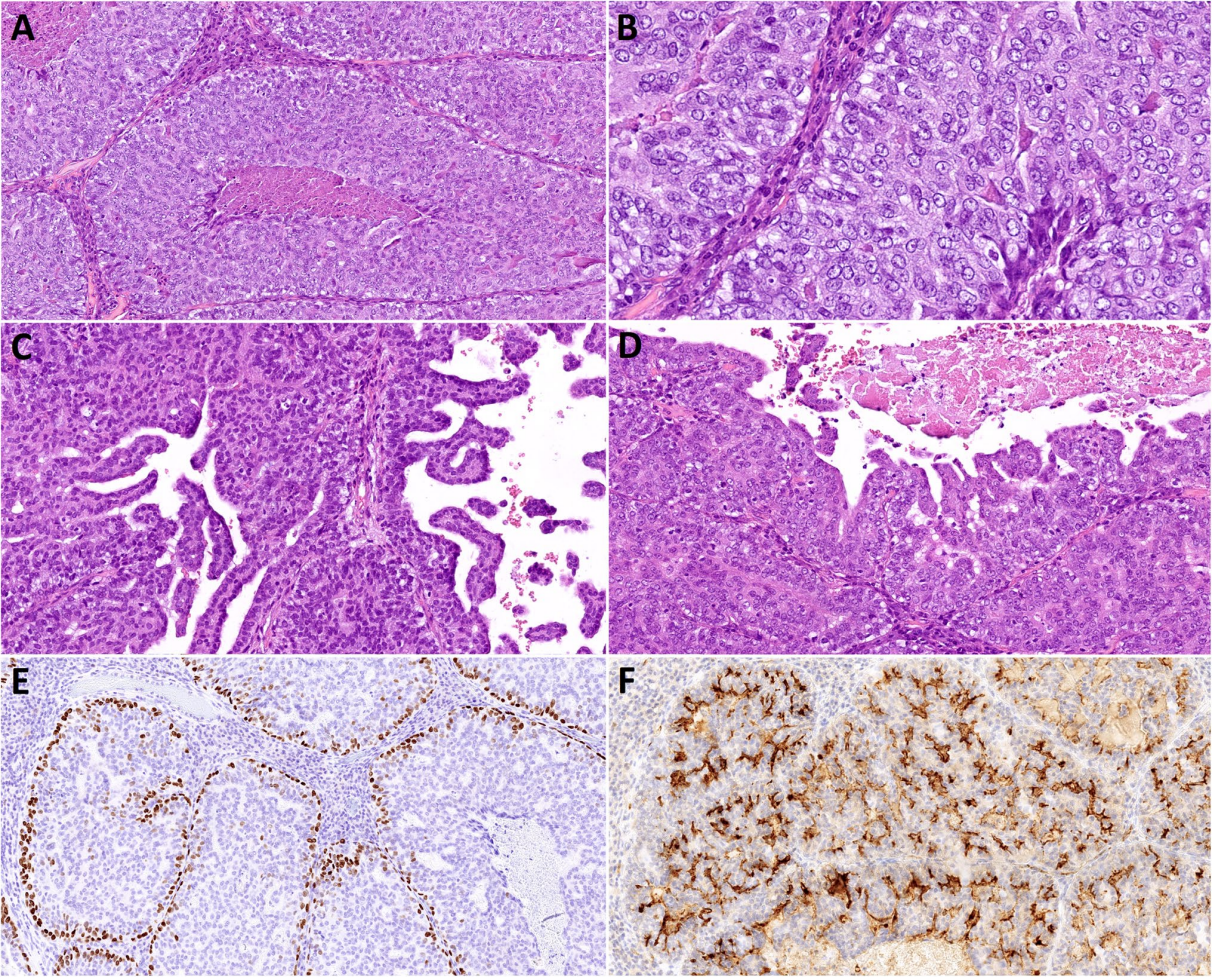
Histopathological features of case 2. **A** The tumor showed features similar to case 1, growing in solid nests with necrotic debris in their centers. **B** Abluminal cells with clear cytoplasm at the periphery of the tumor nests, displaying high-grade cytologic atypia, similar to the luminal tumor cells. **C–D** Cystopapillary, papillary and trabecular architecture was present focally. **E** p63 immunopositivity in the nuclei of the abluminal cells. **F** MUC4 highlighted the membranes of the tumor cells

The tumors widely infiltrated into the submucosa of the nasal cavity. Both tumors displayed high-grade features, growing mostly in hypercellular solid and dense cribriform nests, often with central necrosis (Figs. 2A-B, 3A-B). Minor parts, usually located at the periphery of the lesion, were less dense and showed irregular papillary, microcystic, or trabecular patterns but still displayed high-grade cytological features (Figs. 2A, 3C–D). Crowding of large, oval-to-round, and often vesicular nuclei with prominent eosinophilic nucleoli was observed in both cases. In case 1, the cells contained small or moderate amounts of pale eosinophilic cytoplasm (Fig. 2C–D). In case 2, lining the tumor nests were abluminal cells displaying clear cytoplasmic vacuoles or frank clear-cell change, while the population of luminal cells had pale to moderately eosinophilic cytoplasm (Fig. 3A–B). Numerous apoptotic bodies and brisk mitotic activity (26 and 17 mitotic figures/2.4 mm^2^ in cases 1 and 2, respectively) were observed. Minimal non-neoplastic lymphoplasmacytic infiltration was present in the background in case 1, while case 2 showed more prominent stromal chronic inflammation.

On immunohistochemical examination (Table 1), markers p40 and p63 highlighted the abluminal cells in case 2 (Fig. 3E), similar to cytokeratin 14. These cells corresponded to the abluminal cells with clear intracytoplasmic vacuoles or complete cytoplasmic clearing on H&E slides. In case 1, p63 had the same pattern with nuclear positivity in the abluminal cells lining the tumor nests only in one focus, while in other areas, both p40 and p63 showed randomly scattered nuclear positivity (Fig. 2E). On H&E slides, these cells did not appear different from the p40/p63-negative tumor cells. The tumor cells were diffusely positive for S100, SOX10, and cytokeratins 7, 18, and 19, in both the luminal and abluminal cells. Cytokeratin 8 was only focally positive, while cytokeratin 5/6 was completely negative. PanTrk (Fig. 2F) and GATA3 were diffusely positive in the nuclei of the tumor cells; mammaglobin, however, was negative in both cases. MUC4 was negative in case 1, but it displayed a peculiar pattern of immunostaining in case 2, being positive at the membranes lining the lumina of the small cystic or cribriform formations, as well as a focal membranous positivity in the solid areas of the tumor (Fig. 3F). DOG1 stained the cytoplasmic membranes in 10% of tumor cells in case 1 and was completely negative in case 2 (even after repeated staining). Both tumors showed high proliferative activity, with Ki-67 indices reaching 42% and 51% in cases 1 and 2, respectively.

SMARCB1, SMARCA2, and SMARCA4 immunoexpression was retained in both cases. Smooth muscle actin, androgen receptor, and Her2 were negative in both cases. Furthermore, case 1 did not stain with the SALL4 and TTF1 antibodies, while case 2 was additionally negative for p16, NOR1, CDX2, and STAT6.

### Molecular genetic findings

**Fig. 4:**
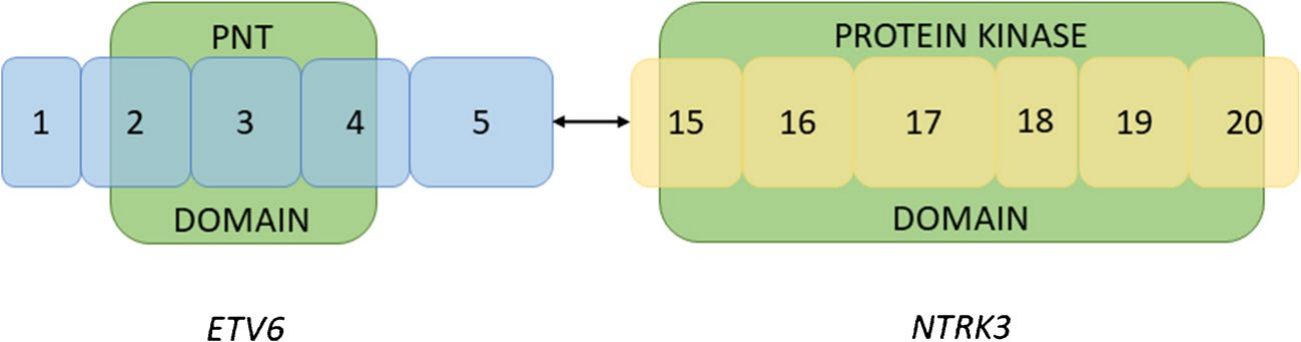
Exon 5 of *ETV6* is fused to exon 15 of *NTRK3*. The fusion product retains the PNT domain of *ETV6*, which enables homodimerization or heterodimerization with other proteins containing the domain, e.g., ETS family proteins. The tyrosine-protein kinase domain of *NTRK3* is also present in the fusion product. This leads to dysregulated activation and constitutive activity of the kinase domain of the fusion protein and, in turn, carcinogenesis

An identical *ETV6::NTRK3* fusion involving exon 5 of the *ETV6* gene and exon 15 of the *NTRK3* gene was detected by RNA-sequencing in both cases (Fig. 4). The reference transcript sequences used for describing *ETV6* and *NTRK3* have accession numbers NM_001987.5 and NM_001012338.3, respectively; the chromosomal position is described using the reference genome GRCh37 (hg19), with breakpoints at chr12:12022900 and chr15:88483984. The NGS-based ligation-dependent multiplex RT-PCR assay corroborated these results. No pathogenic genetic alterations were revealed by the DNA part of the TruSight Oncology 500 panel.

## Discussion

Based on the high-grade morphology with solid, cribriform, and papillary architecture, distinctive immunoprofile with p40/p63, S100, SOX10, and GATA3 positivity, and a complete lack of mammaglobin staining, together with the *ETV6::NTRK3* fusion, we propose the tumors reported herein might represent a subtype of high-grade non-ITAC, distinct from SC.

The major consideration in the differential diagnosis should be a HGT of SC, which was described previously in salivary gland SCs [13]. HGT is defined as the presence of a transformed area lacking the original line of differentiation, consisting of poorly differentiated adenocarcinoma or carcinoma with high-grade features (high mitotic activity and/or necrosis), within an otherwise well-defined, conventional low-grade component characterized by specific microscopic and immunohistochemical features of the particular salivary gland tumor [14]. In contrast to the HGT of SC, no areas with typical SC morphology were identified in our cases. Even though a complete overgrowth of the typical low-grade SC areas by the component with high-grade morphology is possible, this interpretation is unlikely because of the p40/p63 immunoexpression which is not a feature of the high-grade component of SC with HGT. It is therefore more plausible to classify the two cases presented herein as sinonasal high-grade non-ITAC with *ETV6::NTRK3* fusion, analogous to the previously characterized low-grade non-ITACs with this genetic aberration [15–17]. In addition to the analogous morphology and genetic background, these tumors also show a similar immunohistochemical profile. The low-grade tumors are positive for both S-100 and SOX10 or at least one of these markers in the case reported by Rooper et al. [16]. Cytokeratin 7 was positive in all cases. In addition, mammaglobin was negative or showed only focal staining, while GATA3 displayed focal staining in 1/3 cases tested. Conversely, some differences between the low-grade cases reported previously and the high-grade cases presented herein were noted: DOG1 was positive in all of the low-grade cases, but in our study, it showed only limited positivity in one of the two high-grade cases. GATA3 was negative in 2/3 analyzed cases. The markers p40/p63 were tested in only one low-grade case and were negative [16].

Notably, given the tubular and/or cystopapillary architecture and the immunoprofile with S-100, SOX10, and limited DOG1 positivity, origin in the minor salivary glands of the sinonasal tract could be considered in the cases presented herein, as well as the low-grade non-ITAC cases with *ETV6* rearrangement reported previously [15–17], and the tumors could instead be designated “seromucinous salivary gland adenocarcinoma,” perhaps specific for the sinonasal tract. However, such an entity was not included in the current WHO Classification of Tumors, while at present, our cases correspond to the recognized entity of non-ITAC [1]. Further studies may contribute to defining this unit and separating it from the group of non-ITAC.

High-grade adenocarcinoma with similar morphology and an *ETV6::NTRK3* fusion, arising in the parotid gland of a 22-year-old male patient, was reported recently [19]. The tumor exhibited infiltrative solid-papillary and focally glomeruloid patterns of growth, consisting of large, atypical cells with high mitotic activity and areas of necrosis. Similar to our cases, the tumor cells were diffusely positive for MUC4 and GATA3, while exhibiting focal S100 and panTrk staining. The cell nests were focally lined with CK5/6 and p40 immunopositive abluminal cells. In addition, single cells were positive for androgen receptors, whereas mammaglobin and Her2 were negative. After undergoing surgical removal of the tumor and neck lymph node dissection, the short follow-up period of the patient was uneventful. Even though the morphological features and sparse AR immunopositivity were suggestive of salivary duct carcinoma, the tumor was finally diagnosed as high-grade secretory carcinoma, given its localization in the parotid gland, MUC4 and S100 expression, papillary-cystic morphology, and molecular profile [19].

In the same line, a high-grade salivary gland tumor composed of expansile, centrally necrotic nests composed of AR-positive and S100-negative apocrine-type cells with an *ETV6::NTRK3* fusion was recently interpreted as salivary duct carcinoma with an unusual genetic background [20]. This finding is clinically highly relevant, given the poor prognosis and limited treatment options for the entity.

In addition, tumors of a similar morphology, with solid, focally necrotic, as well as micropapillary and cribriform areas, can be noted in previous works that did not include molecular-genetic analysis [22, 23]. However, the morphologically similar tumors showing the poorly differentiated/undifferentiated phenotype reported by Stelow et al. [23] had an immunophenotype somewhat different from our cases: S-100 was negative, and only one of the two tested tumors was positive for CK7, while p63 expression was not tested in this study. Interestingly, some of the high-grade cases reported by Purgina et al. [22] showed a so-called seromucinous immunophenotype, i.e., the expression of at least one of the markers S-100, SOX10, and DOG1, thus approaching the subgroup of *ETV6*-rearranged non-ITACs discussed above.

In summary, we report 2 cases of a high-grade tumor diagnosed as non-ITAC of the sinonasal region, characterized by overt hypercellularity, largely solid growth pattern with comedo-like necrosis, immunohistochemical positivity for p40/p63, S100, SOX10, and GATA3, with a recurrent *ETV6::NTRK3* fusion. Even though they share a common *ETV6::NTRK3* fusion, these high-grade adenocarcinomas might represent a neoplasm distinct from SC by morphology and immunoprofile.

## Data Availability

Data supporting the findings of this study are available within the article. The complete datasets generated during and/or analyzed during the current study are available from the corresponding author upon reasonable request.
